# GelMA-MXene hydrogel nerve conduits with microgrooves for spinal cord injury repair

**DOI:** 10.1186/s12951-022-01669-2

**Published:** 2022-10-28

**Authors:** Jiaying Cai, Hui Zhang, Yangnan Hu, Zhichun Huang, Yan Wang, Yu Xia, Xiaoyan Chen, Jiamin Guo, Hong Cheng, Lin Xia, Weicheng Lu, Chen Zhang, Jingdun Xie, Huan Wang, Renjie Chai

**Affiliations:** 1 State Key Laboratory of Bioelectronics, Department of Otolaryngology Head and Neck Surgery, Zhongda Hospital, School of Life Sciences and Technology, Advanced Institute for Life and Health, Jiangsu Province High Tech Key Laboratory for Bio-Medical Research, Southeast University, Nanjing, 210096 China; 2 The Eighth Affiliated Hospital of Sun Yat-Sen University, Shenzhen, 518033 China; 3 Department of Otolaryngology Head and Neck Surgery, Affiliated Drum Tower Hospital of Nanjing University Medical School, Nanjing, 210008 China; 4 Department of Otolaryngology Head and Neck Surgery, Sichuan Provincial People’s Hospital, University of Electronic Science and Technology of China, Chengdu, 610072 China; 5 Co-Innovation Center of Neuroregeneration, Nantong University, Nantong, 226001 China; 6 Institute for Stem Cell and Regeneration, Chinese Academy of Science, Beijing, 100086 China; 7 Department of Anesthesiology, Sun Yat-Sen University Cancer Center, State Key Laboratory of Oncology in Southern China, Collaborative Innovation for Cancer Medicine, Guangzhou, 510060 Guangdong China; 8 Beijing Key Laboratory of Neural Regeneration and Repair, Capital Medical University, Beijing, 100069 China; 9 Chien-Shiung Wu College, Southeast university, Nanjing, China

**Keywords:** Conductive hydrogel, MXene, Microgroove structure, Neural stem cells, Spinal cord injury

## Abstract

Repair of spinal cord injury (SCI) depends on microenvironment improvement and the reconnection between injured axons and regenerated neurons. Here, we fabricate a GelMA-MXene hydrogel nerve conduit with electrical conductivity and internal-facing longitudinal grooves and explore its function in SCI repair. It is found that the resultant grooved GelMA-MXene hydrogel could effectively promote the neural stem cells (NSCs) adhesion, directed proliferation and differentiation in vitro. Additionally, when the GelMA-MXene conduit loaded with NSCs (GMN) is implanted into the injured spinal cord site, effective repair capability for the complete transection of SCI was demonstrated. The GMN group shows remarkable nerve recovery and significantly higher BBB scores in comparison to the other groups. Therefore, GMN with the microgroove structure and loaded with NSCs is a promising strategy in treating SCI.

**Supplementary Information:**

The online version contains supplementary material available at 10.1186/s12951-022-01669-2.

## Introduction

Spinal cord injury (SCI) is one of the most severe diseases of the central nervous system, often causing partial or complete loss of the physical function below the injury segment [1, 2]. On this basis, it is of great significance to regenerate the functional neurons and enhance cell orientation to promote the connection between the damaged axons and new neurons, and many strategies have been developed for this purpose [3–5]. Recently, researchers have utilized biological scaffolds to support cells grow and differentiate [6]. Also, nutrilite or exosomes have been confirmed the enhanced cell survival rates when injected at the injury site [7–9]. Among them, tissue engineering has been regarded as one of the most effective methods to guide the cells to grow three-dimensionally (3D) [10, 11]. In particular, when the cells are doped into hydrogels, they can be injected into the injured areas precisely and promote the regeneration of neurons [12]. However, this facile operation lacks ingenious microstructure design, restricting the oriented growth of the regenerated neurons [13]. Furthermore, it is exacerbated by the absence of an electrical environment that plays an important role in nerve cells growth [14]. Therefore, the novel hydrogel system with elaborate structures and suitable electrical properties for effective SCI repair is still sought.

**Fig. 1:**
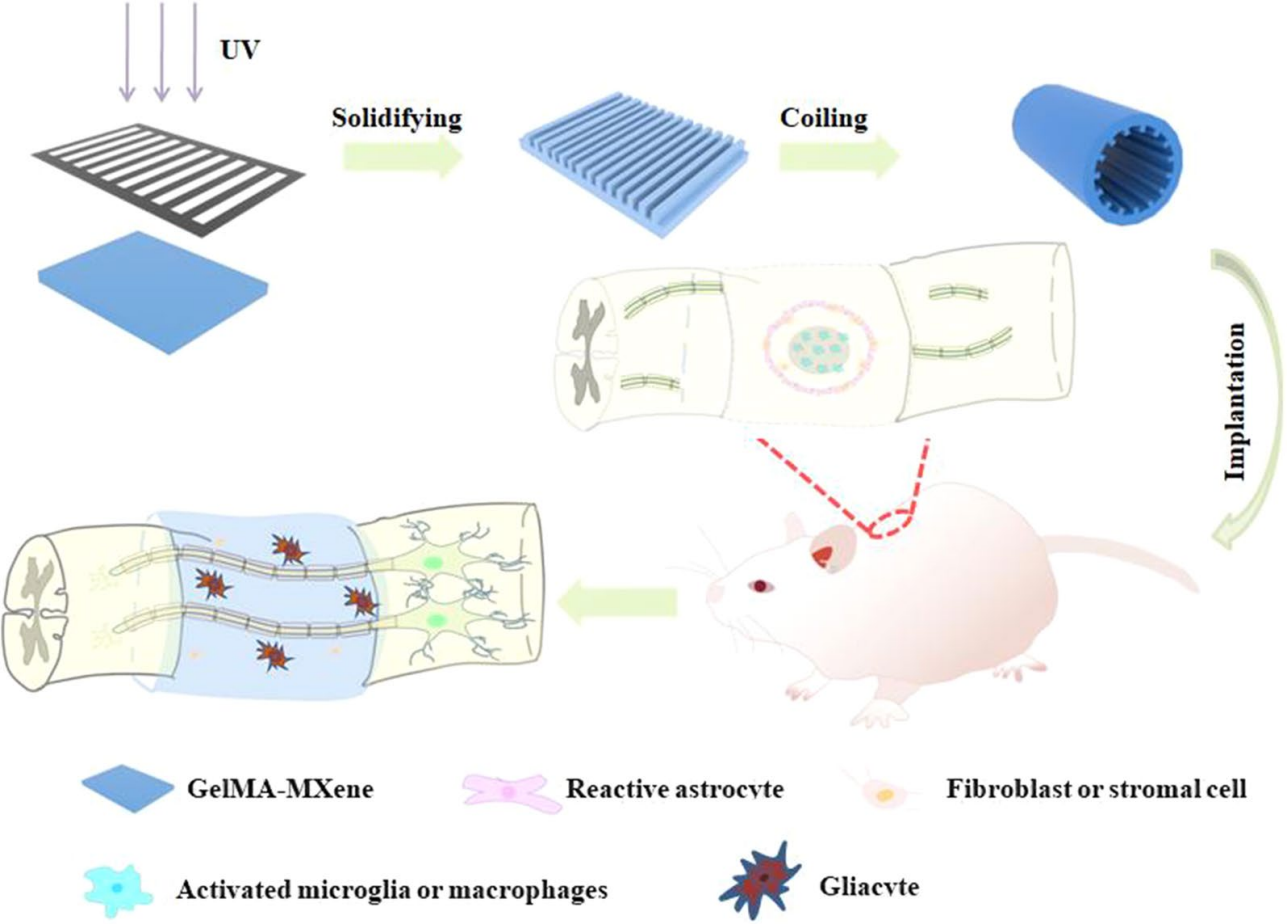
Illustration of fabrication and application of GelMA-MXene hydrogel nerve conduit with microgroove structure

In this work, we developed a conductive MXene-containing hydrogel with a microgroove pattern as a neural guidance conduit to induce nerve cell differentiation and regeneration, as schemed in Fig. 1. MXene is a kind of emerging two-dimensional (2D) nanomaterial with outstanding conductivity and has exhibited huge potential in biomedical applications [15–17], particularly for the treatment of SCI. Besides, MXene possesses excellent hydrophilicity and stability to establish multifunction material [17–19]. At the same time, it has been reported that MXene can be completely degraded in rats [20]. Especially, when MXene is integrated with hydrogels, the resultant composite materials can provide a biocompatible environment and remarkable conductivity for electrical signal transmission among nerve cells [21, 22]. However, most of the developed MXene-doped hydrogels still suffer from poor capability in guiding cell orientation.

Thus, we designed a methylacrylylated gelatin (GelMA) and MXene composite grooved hydrogel film to achieve directional neuron outgrowth. The hydrogel film was prepared by mixing MXene and GelMA solution and polymerized with a grooved mask underultraviolet (UV) light irradiation [23, 24]. GelMA hydrogel we chose in this assay is considered to be one of the hydrogels with excellent biocompatibility that can promote cell growth and proliferation [25, 26]. As a result, the grooved hydrogel was endowed with the outstanding biocompatibility provided by GelMA for cell cultivation and the excellent conductivity by the doped MXene. Additionally, the unique microgroove structure could induce nerve cells to grow directionally and further promote impaired nerve gaps bridge [27–30]. It was demonstrated through in vivo experiments that the implanted nerve guidance conduit effectively promoted the regeneration of the spinal cord and recovery of hind limb motor function in rats. These features indicated that the GelMA-MXene complex functional conduit with microgroove structures have great potential in clinical SCI treatment.

## Experimental section

### Preparation of the GelMA hydrogel films with microgrooves

Methacrylate was not added slowly in the solution until Gelatin (10 g, from porcine skin) was dissolved in PBS (100 mL) at 60 ℃. After the reaction was completed, 400 mL PBS was added and the resultant solution was infused into the dialysis tube (80 cm), and then dialysis was performed in a water bath at 45 ℃ with stirring for 7 days. Next, the sample was frozen by a freeze-drying machine to obtain the solid GelMA. The GelMA pre-gel solution (15 wt%) was prepared by dissolving GelMA in PBS with 2-hydroxy-2-methyl-1-propanone (HMPP, 1 v/v%). To prepare the films, the GelMA pre-gel solution were exposed for 30 s under UV by template sacrificing methods.

### Preparation of the GelMA-MXene hydrogel film with microgrooves

MXene was constructed by etching Ti_3_AlC_2_ (1 g) with hydrofluoric acid (HF). Then Ti_3_AlC_2_ was added at a slow speed. Then the solution was reacted with deionized water via centrifuging after incubating at 40 ℃ for 30 h with stirring. MXene aqueous solution was mixed with GelMA aqueous solution at concentrations of 100, 200, 300, 500 μg/mL, respectively. Finally, different concentrations of GelMA-MXene hydrogel films with grooves were prepared by the photomask.

### Characterizations

The sample was transferred to a freeze dryer at − 50 ℃ for 24 h after being frozen at − 20 ℃. Take the sample into the vacuum chamber of the sputtering instrument and start sputtering 6 times, each time continuing 10 s. At last, the microgroove structures of the sample were observed via using scanning electron microscopy (SEM, Hitachi, S-3000N). The two films’ physical properties, including the detection of swelling ratio, degradation ratio, and compressibility, were measured. The swelling ratio was that the hydrogel was incubated in PBS at 37 ℃ for 10 h after drying, and the solution on the hydrogel surface was removed every other day to measure the mass of the expanded hydrogel. The swelling ratio is calculated as follows:

1 $$\mathrm{Swelling\, ratio }(\mathrm{\%}) =\frac{\mathrm{Ms}-\mathrm{Mi}}{\mathrm{Mi}}\times 100\mathrm{\%}$$

where Ms is the weight of the hydrogel under expansion and Mi is the weight of the initial hydrogel.

The degradation ratio was calculated via the weight of hydrogels degraded at different time points during the 37 ℃ in 0.5 μg/mL collagenase II solution. The formula to calculate the degradation ratio is that

2 $$\mathrm{Degradation \,ratio }(\mathrm{\%}) =\frac{\mathrm{Ws}-\mathrm{Wi}}{\mathrm{Ws}}\times 100\mathrm{\%}$$

where Ws is the dry weight of the initial hydrogel and Wi is the dry weight at each time point.

Modulus of compressibility was determined while the samples were prepared.

### Cell culture

NSCs were derived from the hippocampi of FVB (Friend Virus B) mice (E16-E18). After washed by PBS, the hippocampi were in digestion with accutase solution (Stem cell, USA). Then, the hippocampi were rewashed with PBS, and the proliferation medium was added. After that, the hippocampi were blown into single cells and transferred in flasks, then cultured under 37 ℃ with 5% CO_2_. The proliferation medium consisted of DMEM-F12 (Gibco, USA) with B-27 supplemented (Stem cell, USA), FGF-2 (Life, USA) and EGF (Life, USA). After the passage to the third generation, 7*10^4^ cells/well were seeded on the grooved GelMA hydrogel and GelMA-MXene hydrogel films in 24-well plate. As to differentiation culture, the medium was NeuroCult™ Differentiation Kit (Mouse) (Stem Cell, Canada).

### Cytotoxicity assays

Culturing NSCs on different substrates for 3 days, and the cytotoxicity of various substrates was investigated by Cell Counting Kit-8 (CCK-8, Beyotime). Briefly, GelMA-MXene hydrogel films with different concentrations of MXene were prepared, then NSCs were implanted on these films culturing for 3 days. Next, CCK-8 solution with proliferation medium (1:20) was added to each well after removing the old medium, and then the cells were cultured in heated incubators for 1 h. Finally, the microplate reader was utilized to detect the absorbance at 450 nm of different samples.

### Proliferation experiment

EdU assay was executed by the Click-iT EdU Imaging Kit (Invitrogen, USA). As shown in the protocol, EdU component A was added to the medium and incubated with NSCs at 37 ℃ overnight. Then the cells were fixed in 4% paraformaldehyde (PFA) solution. Click-iT reaction buffer was added to stain the cells at room temperature for 60 min. Zeiss laser scanning confocal microscopy (LSM 700) was used for imaging.

### Immunostaining fluorescence assay

After culture, cells under different treatment conditions were fixed with 4% PFA for 1 h. Next, rinsing cells thrice with PBST (Phosphate buffer solution tween) and then blocking cells in blocking solution for 1 h. After that, cells were stained with primary antibodies including moues anti-nestin, mouse anti-Tuj1 and rabbit anti-GFAP overnight at 4 °C. After that, cells were washed with 0.1% PBST three times and then incubated with appropriate secondary antibody such as Donkey anti-mouse (Alexa Fluor 594, Abcam) or Donkey anti-rabbit (Alexa Fluor 488, Abcam) and DAPI for 1 h. Finally, covering the cells on various substrates via coverslips.

### RT-qPCR

Total RNA was extracted from the NSCs on different substrates via Rneasy Micro Kit (Qiagen). RNA from NSCs cultured on different substrates in proliferation medium for 3 days and differentiation medium for 3 days were extracted respectively to verify the effects of different substrates on the proliferation, adhesion and differentiation of NSCs. The extracted RNA was reversely transcribed into cDNA. The cDNA concentration was then diluted to 500 ng/μL, ultimately ensuring a cDNA concentration of 1000 ng per tube. For quantitative real-time PCR assay, cDNA was added in the mixture of Fast Start Universal SYBR Green Master Mix (Roche), primers, and Rnase-Free Water. PCR program was conducted according to the protocol. PCR quantitative assays were performed in triplicate on each cDNA sample. All primers were purchased from Genscript Biotech Corporation and the sequences of the primers are listed below.

GAPDH-F: AGGTCGGTGTGAACGGATTTG

GAPDH-R: TGTAGACCATGTAGTTGAGGTCA

Nestin-F: CAGCGTTGCAACAGAGGTTGG

Nestin-R: TGGCACAGGTGTCTCAACGGTAG

Nanog-F: CCGGTCAAGAAACAGAAGACCAGA

Nanog-R: CCATTGCTATTCTTCGGCCAGTTG

Sox2-F: TCAGGAGTTGTCAAGGCAGAGAAG

Sox 2-R: GCCGCCGCCGATGATTGTTATTAT

PCNA-F: TTTGAGGCACGCCTGATCC

PCNA-R: GGAGACGTGAGACGAGTCCAT

FAK-F: CCACAGTCTTTGTTCTGGTAGC

FAK-R: CACAAGTTCCAAAACTGCGTG

Myo-10-F: TCCAGACAGACTATGGGCAGG

Myo-10-R: GGAAGCCATGTCGTCCACG

Paxillin-F: GGAGTCTACCACCTCCCACA

Paxillin-R: CCACTGGTCTAAGGGGTCAA

Vinculin-F: TGGACGGCAAAGCCATTCC

Vinculin-R: GCTGGTGGCATATCTCTCTTCAG

MAP2-F: GCCAGCCTCAGAACAAACAG

MAP2-R: AAGGTCTTGGGAGGGAAGAAC

PSD95-F: TGAGATCAGTCATAGCAGCTACT

PSD95-R: CTTCCTCCCCTAGCAGGTCC

Oct4-F: AGCCGACAACAATGAGAACC

Oct4-R: TGATTGGCGATGTGAGTGAT

GFAP-F: TGGCCACCAGTAACATGCAA

GFAP-R: CAGTTGGCGGCGATAGTCAT

### Calcium image

After the proliferation of NSCs for 3 days, FLUO-AM4 staining was added. F-127 powder was mixed into DMSO to prepare 20% (w/v) mother liquor, which was heated and dissolved at 40–50 ℃ for 20–30 min. After the dissolution, it was mixed with the fluorescent probe at a ratio of 1:1, and then the mother liquor of fluorescent probe (solution B) was prepared. The concentration of the working solution was obtained by using phenol red-free DMEM medium with 1:1000 dilution of the solution B. The working solution was incubated for 6–8 min, then washed with PBS thrice. Phenol red-free DMEM solution was added and photographed. The images were obtained by ZEISS confocal microscope (LSM710) equipped with water objective to image living cells. The calcium images were collected every 299.7 ms for 500 cycles. Single-cell ROI was analysed using Image J software and divided by the background ROI normalization. Then, the GraphPad software was used to create an F mark for each cell.

### Surgery for spinal cord transaction and implantation

According to the previous investigation, this study intends to establish the complete transection SCI model of rats. All experimental rats were purchased from Qinglong Mountain. First, Sprague Dawley (SD) rats (male, 350–400 g) were anesthetized with pentobarbital (50 mg/kg) via intraperitoneal injection. After confirming that there was no reaction of pinching the tail and claws, the hair in the lower chest area of the back was removed and disinfected with iodine volt. The skin and muscles were opened with a No. 10 blade to expose the T9–T11 vertebral body. Then, a laminectomy was performed at T9–10 to expose the spinal marrow, and a 2 mm cross-section of the spinal cord was obtained. After that, a hemostatic sponge was temporarily placed in the intersection to control bleeding. The cross-section was washed with PBS, then the material and cells were implanted, and the excess PBS was removed. After surgery, manual urination was performed twice a day. After 2 weeks, reduce it to once a day. All rats were intraperitoneally injected with penicillin for 1 week. All the rats were divided into the control group (control), GelMA-NSC group (GN), GelMA-MXene-NSC group (GMN). All animal schemes received confirmation from the Animal Experimental Ethical Inspection Committee of Southeast University (No. 20, 210, 401, 009).

### Assessment of motor function and urination function

Behavioral outcomes were evaluated at 1, 2, 3, 4, 5, 6, 7, 8 weeks postoperatively. A BBB exercise rating scale was administered to each animal in the treatment groups by two random observers. In addition, with the help of video recording, the movement state of each rat was observed. Tissues of the bladder were collected and the weight was recorded to verify the remediation effect of the conductive hydrogel.

### Immunohistochemistry

At the end of the treatment trial, the animals were instilled with isotonic physiological saline to be deep anesthesia. Spinal cord tissue was taken about 2 cm around the lesion site. The tissue was embedded and frozen into 10 μm sections by cryotomy. After being fixed with 4% PFA at 4 ℃, the sections were incubated with mouse anti-Tuj1, rabbit anti-GFAP, rabbit anti-NF and rabbit anti-Oligo2 overnight. After washing via 0.1% PBST, the samples were incubated with Alexa Fluor 488 or 555 antibodies and DAPI (labeled nucleus) at room temperature for 1 h. Laser scanning confocal microscopy (LSM700) completed observation of the sample.

### Statistical analysis

In this experiment, unless otherwise specified, three replicates were set for all the experimental groups, and the statistical data were determined by the standard error of mean. P < 0.05 indicated a significant difference. Image J was used to process statistical data, and Graphpad Prism software was used for drawing and data analysis. Image Pro Plus was applied to analyze the direction of the cells on different substrates.

## Results and discussion

### Characterization of grooved GelMA-MXene hydrogels

Koti et al. have proved that 15% GelMA most significantly improves cell survival rate, so we chose this concentration in our study [31]. Firstly, to determine the cytotoxicity of MXene, CCK8 was used to perform toxicity tests on cells cultured on different substrates for 3 days. It was found that the concentrations of MXene up to 500 μg/mL did not affect cell growth (Additional file 1: Fig. S1a). Next, we explored the influence of different concentrations of MXene on NSCs proliferation with EdU (5-ethynyl-2′-deoxyuridine) assay. Additional file 1: Fig. S1b shows that the concentration at 100 and 200 μg/mL of MXene observably promote the proliferation of NSCs, while the higher concentration exhibited adverse impacts. Considering such results and the conductivity of different concentrations of GelMA-MXene groove membrane, the final concentration of MXene was determined to be 200 μg/mL (Additional file 1: Fig. S2a).

**Fig. 2:**
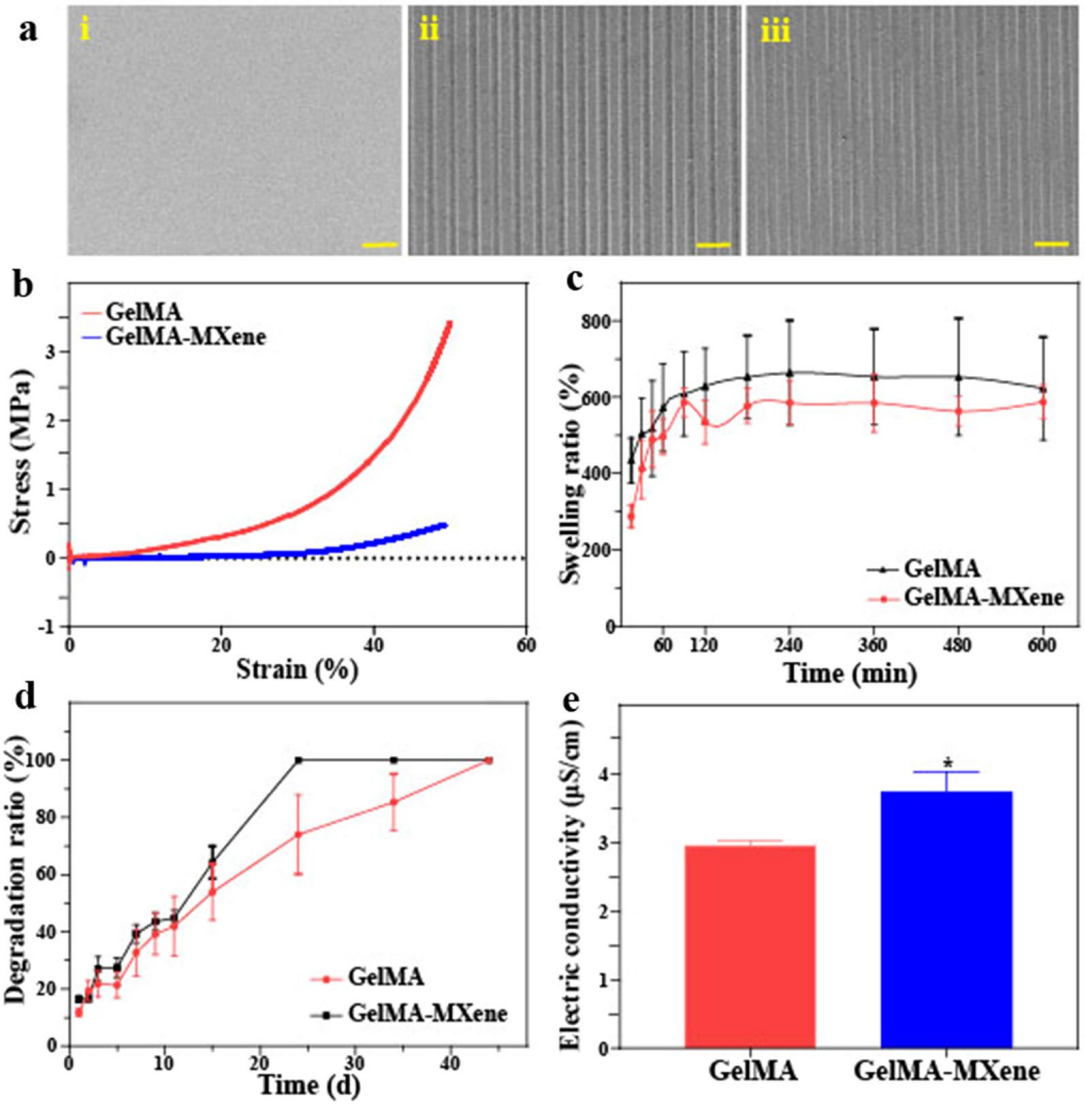
Characterization of GelMA-MXene hydrogel. **a** SEM images of TCPs (i), GelMA substrate (ii), and GelMA-MXene substrate (iii), respectively. Scale bars: 100 μm. **b**–**e** The compression property (**b**), swelling performance (**c**), degradation performance (**d**), and conductivity € of the two kinds of hydrogels. *p < 0.05, **p < 0.01, ***p < 0.001

Then, GelMA-MXene film with microgroove structures was prepared by photomask etching, and their morphology was characterized via using scanning electron microscopy (SEM), which was shown in Fig. 2a. It was found that compared with the pure tissue culture polystyrene (TCP), either GelMA or GelMA-MXene films were endowed with the oriented groove structures with a width of 20 μm. Previous studies have shown that grooves in the range of 1.5 – 20 μm are conducive to directional cell growth, and the wider grooves could increase the adhesion of cells [32, 33]. To investigate the mechanical performance of the prepared GelMA-MXene hybrid hydrogel, the compression deformation test and cyclic compression test were conducted. The compressive force of GelMA hydrogel was in 0–3.4 Mpa, while the compressive strength of GelMA-MXene hydrogel was in the range of 0–0.47 Mpa, both of which were proved suitable as tissue engineering scaffold (Fig. 2b) [34, 35]. The main reason resulting in this difference may be that the light impermeability of MXene affected the GelMA hydrogel crosslinking to a certain extent. Particularly, to apply the resultant hydrogel to the SCI repair, grooved GelMA and GelMA-MXene hydrogel films with a diameter of 5 mm were prepared and then rolled into conduits for in vivo nerve bridge (Additional file 1: Fig. S3a and b). The mechanical strength and stability of such two nerve conduits were confirmed by compression cycle tests (Additional file 1: Fig. S3c and d).

It was found that GelMA and GelMA-MXene hydrogels achieved swelling equilibrium after 10 h, and the swelling ratio of GelMA-MXene hybrid hydrogel reached around six times, which was beneficial to the in vivo substance exchange occurrence (Fig. 2c). Next, in order to determine the degradability of the two hydrogels, we detected their degradation behavior in 0.5 μg/mL collagenase solution. It was observed that both pure GelMA and GelMA-MXene hybrid hydrogel showed a slow degradation rate and complete degradation to provide sustained physical support for defected nerves, demonstrating the feasibility of in vivo application (Fig. 2d). Benefitting from the integration of MXene, the electrical conductivity of GelMA-MXene hybrid hydrogel was stable at 3.6 μS/cm and significantly higher than that of pure GelMA hydrogel, as recorded in Fig. 2e.

### Adhesion and directions of NSCs on grooved GelMA-MXene hydrogels

**Fig. 3:**
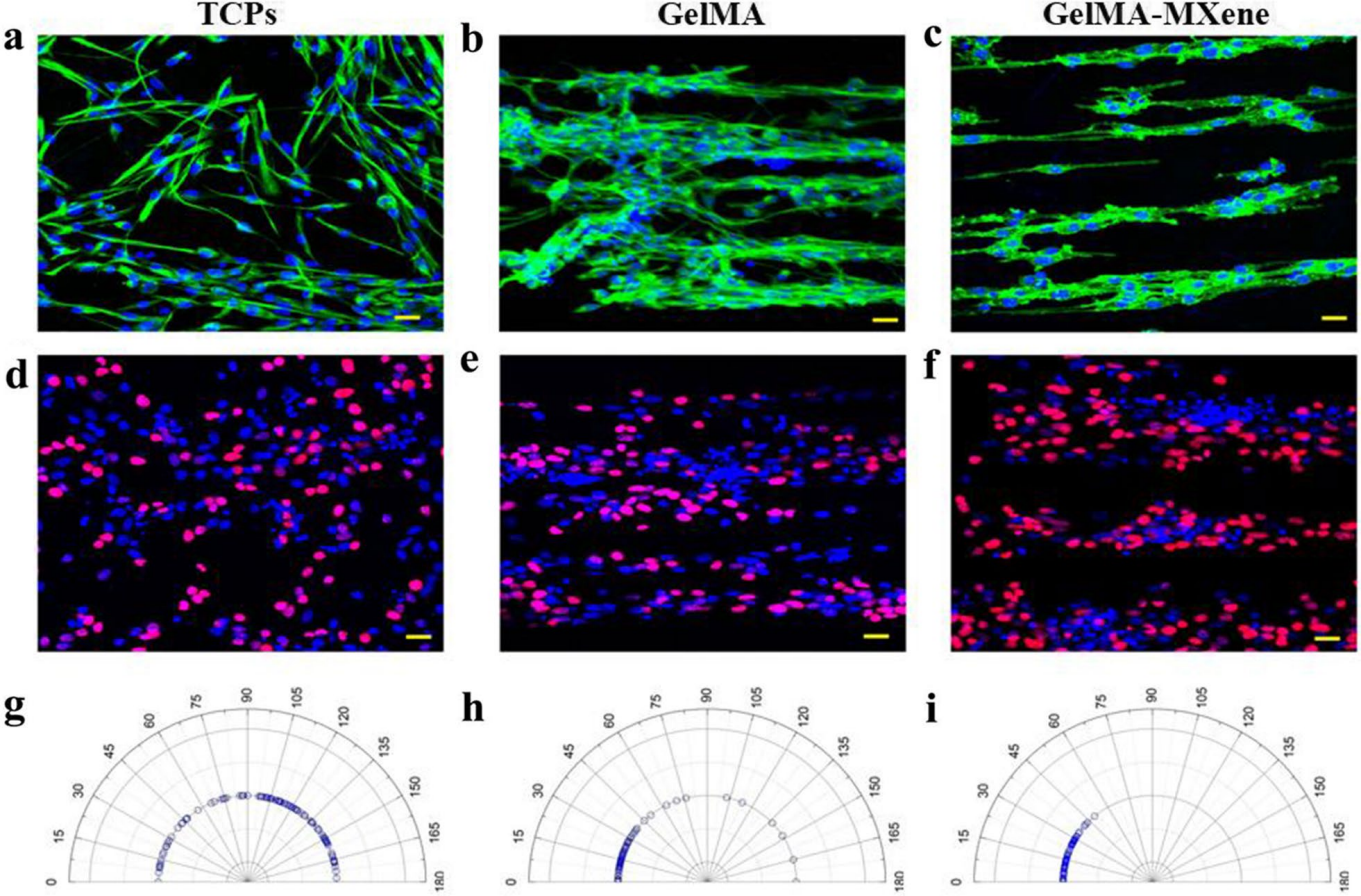
Biocompatibility and proliferation of GelMA-MXene hydrogel membrane for NSCs. **a**–**c** Immunofluorescence staining images of NSCs planted on TCPs (**a**), grooved GelMA film (**b**), and grooved GelMA-MXene film (**c**), respectively. Marking Nestin in green, nuclei in blue. Scale bars: 20 μm. **d**–**f** Immunofluorescence staining images of NSCs planted on TCPs (**d**), grooved GelMA fi€ (**e**), and grooved GelMA-MXene film (**f**), respectively. EdU was marked in red, and nuclei in blue. Scale bars: 20 μm. **g**–**i** Directional statistics of NSCs cultured on TCPs (**g**), grooved GelMA film (**h**), and grooved GelMA-MXene film (**i**), respectively

Biotoxicity of materials has great significance in their biomedical application. Thus, we verified the toxic effects of three different substrates on NSCs, and it was found that none of these substrates would harm the survival of NSCs (Additional file 1: Fig. S4a). In particular, it was observed that the groove structure could promote the directional arrangement of NSCs and significantly increase local adhesion [36]. Nestin-labeled immunofluorescence showed that NSCs were aligned and grew along the groove on both GelMA hydrogel and GelMA-MXene hydrogel, while NSCs on TCPs showed disordered growth (Fig. 3a–c). The Real-time quantitative PCR (RT-qPCR) results turned out that the expressions of cell adhesion factors FAK, Myo-10, Paxillin, and Vinculin in these two hydrogel grooves were obviously higher than those on TCPs (Additional file 1: Fig. S4b). This enhanced local adhesion could be ascribed to the fact that grooves provide more sites for cells adhesion.

### Proliferation of NSCs on grooved GelMA-MXene hydrogels

Besides, NSCs were inoculated on the three substrates and incubated in the medium for 3 days to evaluate the effect of grooved hydrogel film on NSCs proliferation. EdU immunofluorescence figures and relevant statistical analysis results show that both GelMA and GelMA-MXene hydrogel membranes facilitated the directed proliferation of NSCs compared to the TCPs group (Fig. 3d–f, and Additional file 1: Fig. S4c). Similarly, the orientation of NSCs on different substrates was statistically analyzed, which showed that NSCs planted on the groove membranes were concentrated at a specific angle, while those grown on TCPs grew in all directions (Fig. 3g–i). These results confirmed that the groove structure effectively promoted the directional growth of cells, consistent with the results in Fig. 3a–c. Furthermore, RNA expressed by NSCs cultured on different substrates was extracted and reverse-transcribed into cDNA after proliferation for 3 days. The mRNA expressions of proliferation-associated genes, including Nanog, Nestin, PCNA, Sox2, and Telomerase (TE) genes, were analyzed by RT-qPCR technology. The result revealed that both kinds of grooved hydrogel films enhanced the mRNA expression of Nanog, Nestin, PCNA, Sox2, and TE genes compared to the TCPs group, which could be credited to the grooved GelMA hydrogel (Additional file 1: Fig. S4d).

### Directional differentiation of NSCs on grooved GelMA-MXene films

**Fig. 4:**
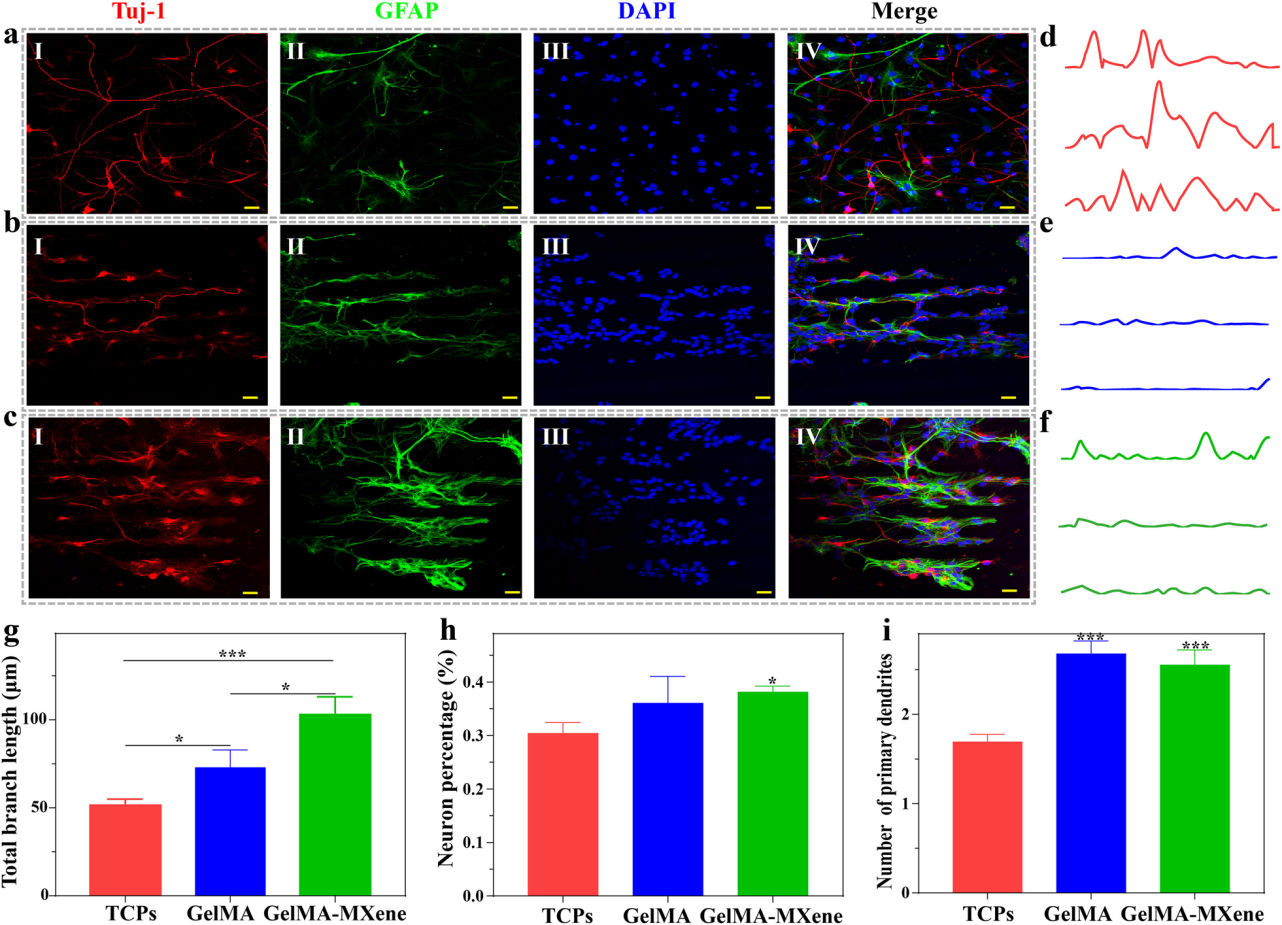
The differentiation of NSCs on grooved GelMA-MXene hydrogel. **a**–**c** Immunofluorescence staining images of NSCs seeded on TCPs (**a**), grooved GelMA (**b**) and grooved GelMA-MXene hydrogel (**c**) for 3 days. Tuj-1 was stained red, GFAP was stained in green, and nuclei were stained blue. Scale bars: 20 μm. **d**–**f** Curvature analysis of NSCs on TCPs (**d**), grooved GelMA hydrogel (**e**) and GelMA-MXene hydrogel (**f**). **g**–**i** The total length of the neurites (**g**), the differentiation rate of NSCs (**h**), and the number of primary branch tips (**i**) of the three types of substrates. *p < 0.05,   *p < 0.01, ***p < 0.001, and ns mark means no significant difference

NSCs were planted on different substrates (TCPs, grooved GelMA and GelMA-MXene films) and cultured in the differentiation medium for 3 days, and the differentiation and arrangement of NSCs were analyzed by immunofluorescence staining, as shown in Fig. 4a–c. It was observed that NSCs successfully differentiate into neurons (labeled with βIII-tubulin, Tuj-1) and glial cells (labeled with glial fibrillary acidic protein, GFAP) on three kinds of substrates and grew directionally on the grooved hydrogel films. Curvature analysis also demonstrated that NSCs cultured on TCPs showed directionless growth, while the growth of NSCs on both films with microgrooves was in direction (Fig. 4d–f). Meanwhile, SEM images confirmed such orientated growth of differentiated neurons along the grooves (Additional file 1: Fig. S5a–c). The total axon length of NSCs differentiated neurons cultured on the GelMA-MXene hydrogels was markedly higher than that on the GelMA hydrogels and TCPs (Fig. 4g). Of note, we discovered the enhanced differentiation of NSCs into neurons on the grooved GelMA-MXene film. In contrast, there was no prominent difference between the GelMA and TCPs groups (Fig. 4h). These properties could be ascribed to the active influence of the brilliant electrical conductivity of MXene on the differentiation of NSCs. In terms of the number of primary branch points of neurons, the two kinds of grooved hydrogels were both advantageous on axon branches (Fig. 4i). This result revealed that the grooved structures provided more adhesion sites for NSCs differentiation.

### Calcium change of NSCs incubated on grooved GelMA-MXene hydrogel film

**Fig. 5:**
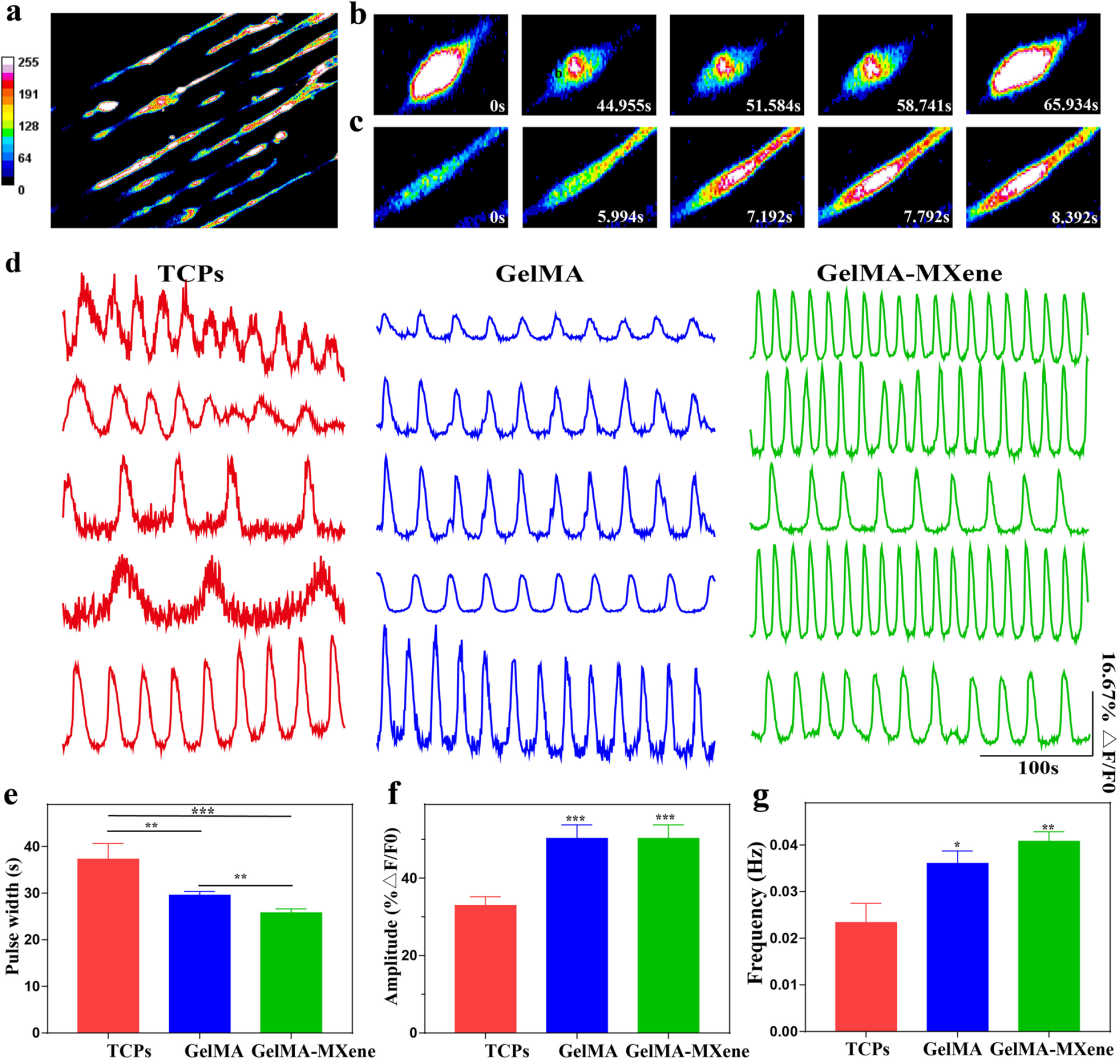
Calcium images of NSCs cultured on three substrates. **a** Representative calcium image of NSCs cultured on GelMA-MXene films. **b**, **c** Ca^2+^ transients of representative individual cell at different time points on grooved GelMA hydrogel films (**b**) and GelMA-MXene hydrogel films (**c**). **d** Representative calcium transient traces of NSCs seeded on three substrates. **e**–**g** Histograms of the pulse width (**e**), the relative fluorescence amplitude (**f**) and the frequency (**g**) of Ca^2+^. *p < 0.05, **p < 0.01, ***p < 0.001 and ns mark means no significant difference

Ca^2+^ regulates the activities of neuronal networks to promote neurons development and maturation [37]. In order to research the survival and function of NSCs seeded on grooved GelMA-MXene hydrogel, Ca^2+^ imaging was performed on NSCs after 3 days of proliferation culture. Calcium images of the NSCs were collected every 299.7 ms for a total of 500 cycles. Figure 5a provided the presentative overview of the Ca^2+^ fluorescence information of NSCs cultured on the GelMA-MXene hydrogels, in which different color reflected the intracellular concentration difference of Ca^2+^. The spontaneous Ca^2+^ peaks of a single NSCs on the grooved GelMA and GelMA-MXene films were shown in Fig. 5b and c, respectively. Figure 5d presented the normalized fluorescence traces of NSCs cultured on three different substrates. The waveform parameters of the normalized fluorescence trace, including amplitude, pulse width and frequency, which reflected the relative amplitude, bursting time and interval reciprocal of intracellular Ca^2+^ peak, were then quantified [38]. The NSCs cultured on the grooved hydrogels showed higher calcium transient frequency and amplitude, comparing with the NSCs seeded on the TCPs (Fig. 5e–g and Additional file 2: Supplement video 1).

### In vivo evaluation of grooved GelMA-MXene hydrogel conduits for SCI repair

**Fig. 6:**
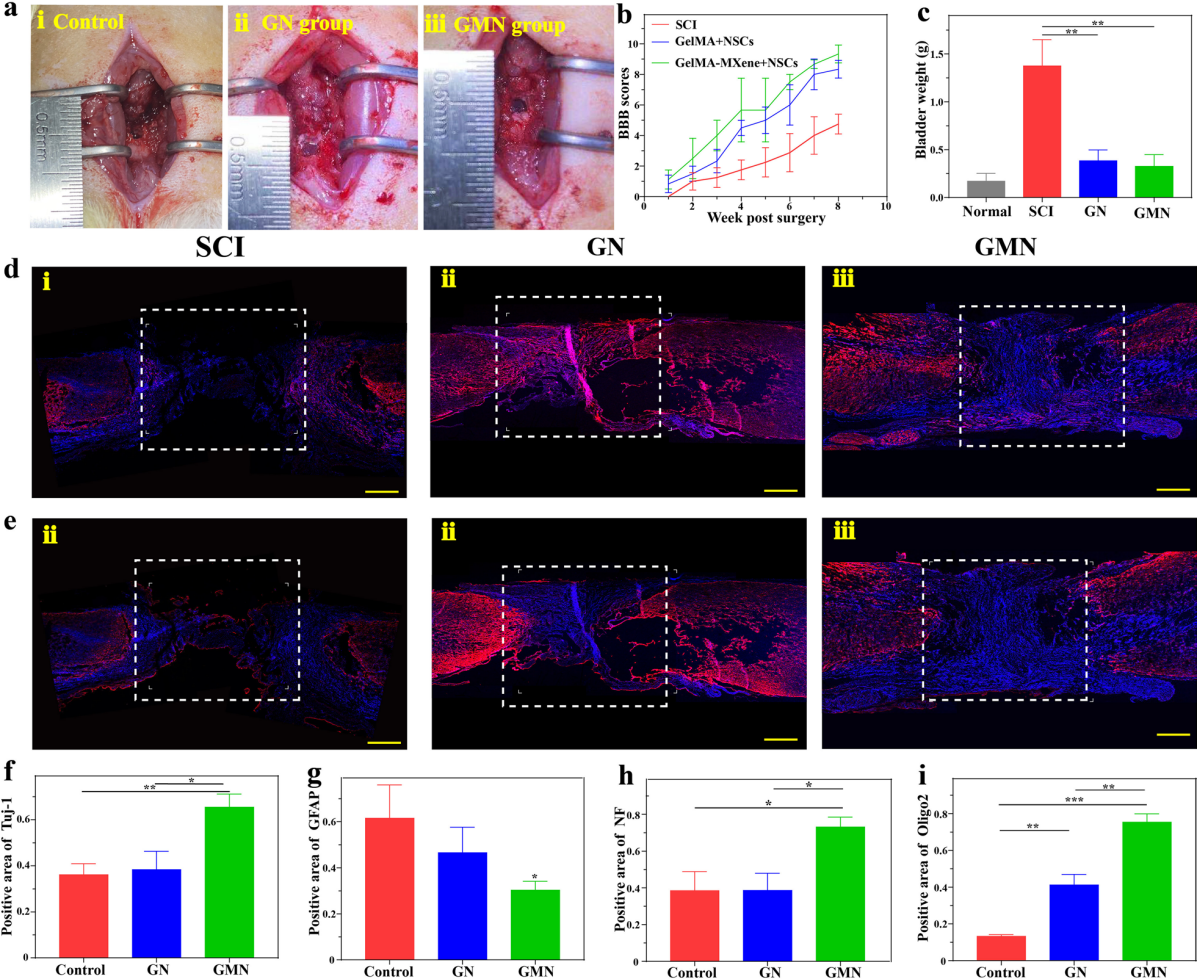
Regeneration of spinal cord tissue on 8 weeks. **a** Representative images of SCI: (i) the exposed spinal cord, (ii) GN implanted, (iii) GMN implanted. **b** BBB scores of rats in control group, GN group, and GMN group. **c** The bladder weight of rats in control group, GN group, and GMN group. **d** Immunofluorescence staining (Tuj-1, red; DAPI, blue): (i) control group, (ii) GN group, (iii) GMN group. **e** Immunofluorescence staining (GFAP, red; DAPI, blue): (i) control groups, (ii) GN groups, (iii) GMN groups. **f**–**i** The positive area of Tuj-1 (**f**), GFAP (**g**), NF (**h**) and Oligo2 (i). *p < 0.05, **p < 0.01, ***p < 0.001 and ns means no significant difference

To explore the feasibility of resultant conduits for SCI repair, rat models with complete transected SCI were constructed. After sterile treatment, NSCs were planted and cultured in the proliferation medium for 1 day, then transferred to the hydrogel films, followed by being curled into the conduits. GelMA-NSCs (GN group) and GelMA-MXene-NSCs (GMN group) were separately implanted into the defected sites of transected spinal cord injured rats, and the rats of control group were receiving PBS treatment at the lesion site (Fig. 6a). To assess the hindlimb locomotion recovery of the animals in different groups, we performed Basso-Beattie-Bresnahan (BBB) test each week, as exhibited in Fig. 6b. It was found that the scores of all the rats were 0 after SCI surgery, demonstrating the injury models were successfully constructed. After implantation for 8 weeks, the BBB scores of the GN group and GMN group were 8.3 ± 0.5 and 9.3 ± 0.5 respectively, which revealed evident differences with the control group (4.6 ± 0.9). The Additional file 2: videos further confirmed that the rats in the GN group and GMN group achieved the apparent hindlimb movements, while there was no obvious movement in the control group. These results showed that integrating exogenous NSCs with grooved hydrogel conduits had a significant effect on nerve regeneration, which might be due to the directional differentiation of NSCs growing on the grooved hydrogels and enhancing the contact with the axons of the host element.

Urinary system disease is one of the complications of SCI [39]. The physical function below the injury segment would be partially or completely lost, leading to irreversible damage of bladder tissues [40]. Thus, the effective recovery of urinary damage is an significant indicator to estimate the therapeutic effect. Figure 6c showed the weight of bladders in the control group increased significantly within 8 weeks after surgery. Meanwhile, the volume of bladders in the GN and GMN groups showed an apparent difference from the control group (Additional file 1: Fig. S6). This result confirmed that GelMA or GelMA-MXene implantation was beneficial to protect the urinary system, indicating the SCI repair effects.

Furthermore, all the rats were finally sacrificed to collect the injured spinal cords at 8 weeks after surgery. Then, to detect differentiation at the injured site, we dyed tissues with Tuj-1 and GFAP antibodies (Fig. 6d and e). It was obvious that there was a large cavity in the control group, while plenty of neurons was observed in the other two groups (Fig. 6d). The expression of Tuj-1 in the GMN group (65.47%) was remarkably higher than that in the control group (36.12%), as shown in Fig. 6f. Meanwhile, quantitative analysis for GFAP demonstrated that the positive-staining area in the GMN group (30.32%) was lower than that in the GN group (46.53%) and control group (66.90%), as indicated in Fig. 6g. In consequence, GelMA-MXene conduit loaded with NSCs took advantage of promoting neuronal differentiation and restraining the formation of glial scars. We also determined the growth of nerve fibers in the lesion area via staining neurofilament (NF). It was found that a large number of newborn NF-positive cells were distributed in the GN and GMN groups, whereas there was barely visible NF-positive cells in the control group (Additional file 1: Fig. S7). In addition, it was found that GMN group promoted more effectively the growth of newborn NF compared with GN group. Next, we chose Oligo2 to dye oligodendrocyte cells at the lesion site. Observably, the positive Oligo2 cells in the GMN group were found throughout the site where conduits were implanted, comparing which in the control and GN groups (Additional file 1: Fig. S7). These results were further demonstrated by quantitative analysis, as shown in Fig. 6h and 6i. In brief, it turned out that GMN group effectively promoted the connection between the newborn nerves with the injured nerve. Lei et al. has proved that the electroconductive hydrogel with bone marrow stem cell-derived exosomes can improve the microenvironment of the injured site [41]. It showed that electroconductive hydrogel also need the good biocompatibility. The injectable and conductive polysaccharides-based hydrogels have been found to promote the differentiation of NSCs into neurons and inhibit astrocyte differentiation in vitro [42]. However, they have not performed experiments on the repair of spinal cord injury. Thus, we thought that GelMA-MXene hydrogel possessed multiple features, such as electricity, biocompatibility and topological property to repair spinal cord injury.

Last, to concern the survival and differentiation of the implanted NSCs, we seeded GFP-NSCs on the GelMA-MXene hydrogel nerve conduit to repair SCI. Additional file 1: Fig. S8a illustrated the colocalization of the GFP and Tuj-1, demonstrating that the exogenous NSCs could survive on the conduit and differentiate into neurons. The GFP-NSCs co-stained with NF further proved the survival and mature neuron formation (Additional file 1: Fig. S8b). Moreover, it was also found that the GFP-positive NSCs were co-stained with Oligo2 antibody, indicating the grafted NSCs could differentiate into oligodendrocytes for nerve regeneration (Additional file 1: Fig. S8c).

## Conclusion

In this article, we developed a multifunctional scaffold containing the conductive composition and topological structure to improve the microenvironment of the injured site in spinal cord and facilitate the connection between the newborn nerve and the injured axis. We constructed a GelMA-MXene hybrid hydrogel film with a microgroove pattern via mask and then rolled it into the tubular hydrogel for subsequent in vivo application. In vitro experiment demonstrated that the grooved GelMA-MXene hydrogel effectively improved the adhesion, proliferation and differentiation of NSCs. Furthermore, through the animal experiment, the feasibility of GMN scaffolds for SCI was explicitly confirmed in vivo. We expect the conductive scaffolds can increase more possibilities for applications in neural tissue engineering.

## Supplementary Information

**Additional file 1: Fig. S1.** Biocompatibility and proliferation of NSCs under different concentrations of MXene. **Fig. S2.** Conductivity of GelMA-MXene hydrogels with different concentrations of MXene. **Fig. S3.** Characterization of the two conduits. **Fig. S4.** Biocompatibility and proliferation of NSCs on different substrates. **Fig. S5.** Apparent morphology NSCs differentiation on the three substrates. **Fig. S6.** The bladders of different groups on week 8. **Fig. S7.** Representative images of spinal cord at the lesion rate. **Fig. S8.** Survival and differentiation of the grafted NSCs in the lesion site four weeks post-implantation.

**Additional file 2: Supporting Video S1.** The action of the rats of different groups at 8 weeks post-implantation.

## Data Availability

The data supporting the conclusions of this article are included within the article and its supplementary information.
