# Supplementary material for: GelMA-MXene hydrogel nerve conduits with microgrooves for spinal cord injury repair
Source: J Nanobiotechnology. 2022 Oct 28;20:460. doi: 10.1186/s12951-022-01669-2 (PMC9617371; doi:10.1186/s12951-022-01669-2)
Supplement: Supplementary file 1 — Additional file 1: Fig. S1. Biocompatibility and proliferation of NSCs under different concentrations of MXene. Fig. S2. Conductivity of GelMA-MXene hydrogels with different concentrations of MXene. Fig. S3. Characterization of the two conduits. Fig. S4. Biocompatibility and proliferation of NSCs on different substrates. Fig. S5. Apparent morphology NSCs differentiation on the three substrates. Fig. S6. The bladders of different groups on week 8. Fig. S7. Representative images of spinal cord at the lesion rate. Fig. S8. Survival and differentiation of the grafted NSCs in the lesion site four weeks post-implantation. [file 12951_2022_1669_MOESM1_ESM.docx]

Supporting information

**GelMA-MXene hydrogel nerve conduits with microgrooves for spinal cord injury repair**

*Jiaying Cai^1,^* ^‡^*, Hui Zhang^1,^* ^‡^*, Yangnan Hu^1,^* ^‡^*, Zhichun Huang^1,^* ^‡^*, Xiaoyan Chen^1^, Jiamin Guo^1^, Hong Cheng^1^, Lin Xia^1^, Chen Zhang^6^, Huan Wang^2,^*, and Renjie Chai^1,3,4,5,6,^**

1 State Key Laboratory of Bioelectronics, Department of Otolaryngology Head and Neck Surgery, Zhongda Hospital, School of Life Sciences and Technology, Advanced Institute for Life and Health, Jiangsu Province High Tech Key Laboratory for Bio-Medical Research, Southeast University, Nanjing 210096, China

2 The Eighth Affiliated Hospital of Sun Yat-Sen University, Shenzhen, 518033, China

3 Department of Otolaryngology Head and Neck Surgery, Affiliated Drum Tower Hospital of Nanjing University Medical School, Nanjing 210008, China

4 Department of Otolaryngology Head and Neck Surgery, Sichuan Provincial People's Hospital, University of Electronic Science and Technology of China, Chengdu 610072, China

5 Co-Innovation Center of Neuroregeneration, Nantong University, Nantong 226001, China; Institute for Stem Cell and Regeneration, Chinese Academy of Science, Beijing 100086, China

6 Beijing Key Laboratory of Neural Regeneration and Repair, Capital Medical University, Beijing 100069, China

Corresponding Author

*Email: [renjiec@seu.edu.cn](mailto:renjiec@seu.edu.cn); [wangh679@mail.sysu.edu.cn](mailto:wangh679@mail.sysu.edu.cn)

‡ These authors contribute equally to this work.

Table of contents:

Figure S1. Biocompatibility and proliferation of NSCs under different concentrations of MXene 2

Figure S2. Conductivity of GelMA-MXene hydrogels with different concentrations of MXene 2

Figure S3. Characterization of the two conduits 3

Figure S4. Biocompatibility and proliferation of NSCs on different substrates 4

Figure S5. Apparent morphology NSCs differentiation on the three substrates 5

Figure S6. The bladders of different groups on week 8 5

Figure S7. Representative images of spinal cord at the lesion rate 5

Figure S8. Survival and differentiation of the grafted NSCs in the lesion site four weeks post-implantation 6

**Supporting Figures**

**
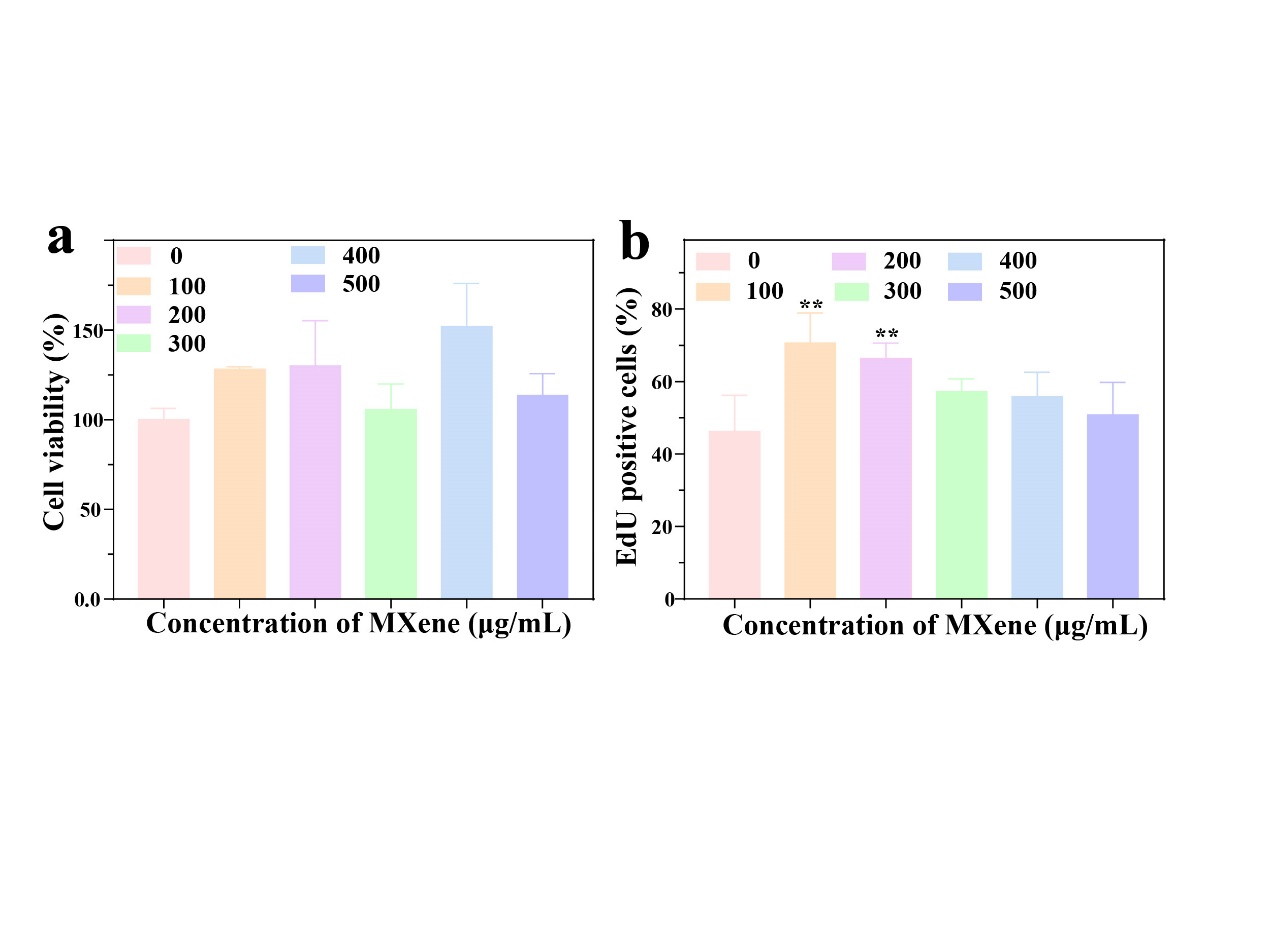
**

**Figure S1.** Biocompatibility and proliferation of NSCs under different concentrations of MXene. (a) Cell viability on the NSCs cultured in different concentrations of MXene (μg/mL), determined by CCK-8 assay. (b) Cell proliferation of NSCs under different concentration of MXene (μg/mL), ∗p *<*0.05, ∗∗p *<*0.01, ∗∗∗p *<*0.001 and ns means no significant difference.

**
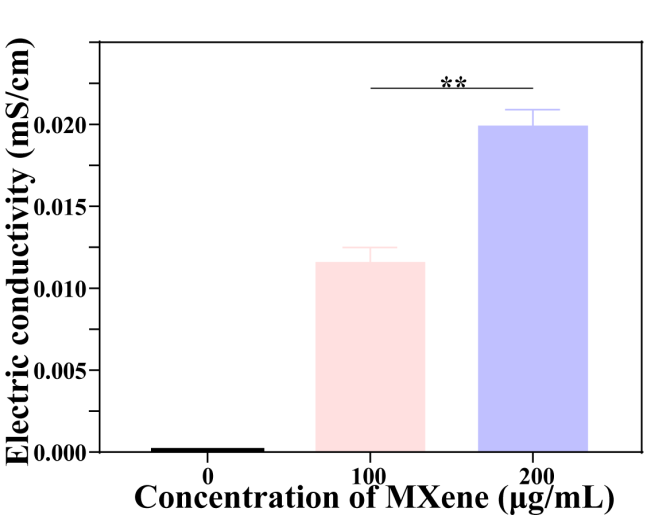
**

**Figure S2.** Conductivity of GelMA-MXene hydrogels with different concentrations of MXene. ∗p *<*0.05, ∗∗p *<*0.01, ∗∗∗p *<*0.001 and ns means no significant difference.

**
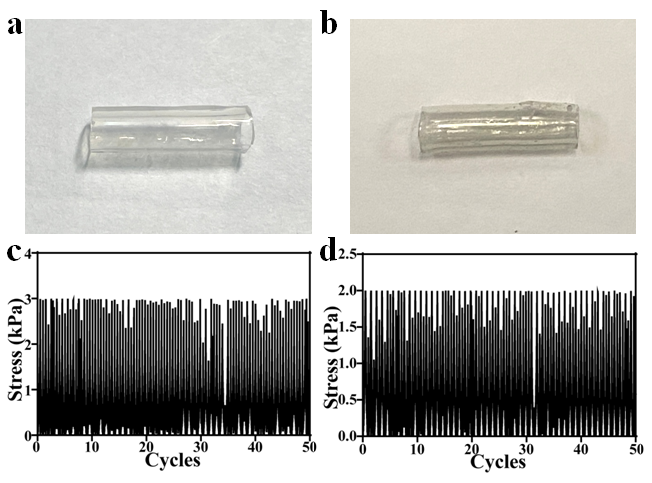
**

**Figure S3.** Characterization of the two conduits. (a) GelMA hydrogel conduit with microgrooves. (b) GelMA-MXene hydrogel conduit with microgrooves. (c) Mechanical cycle test on GelMA hydrogel conduit with microgrooves. (d) Mechanical cycle test on GelMA-MXene hydrogel conduit with microgrooves.

**
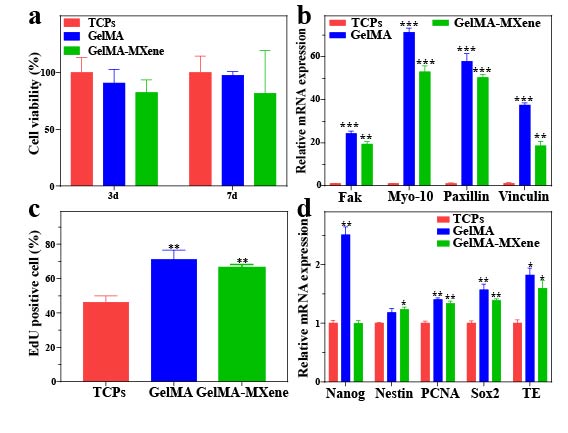
**

**Figure S4.** Biocompatibility and proliferation of NSCs on different substrates. (a) Cell viability on the NSCs cultured on TCPs, grooved GelMA hydrogel films and GelMA-MXene hydrogel films, determined by CCK-8 assay. (b) Cell adhesion-related gene expression of NSCs seeded on TCPs, grooved GelMA hydrogel films and GelMA-MXene hydrogel films, determined by RT-qPCR assay. (c) Cell proliferation of NSCs on TCPs, grooved GelMA hydrogel films and GelMA-MXene hydrogel films. (d) Cell proliferation-related gene expression of NSCs seeded on TCPs, grooved GelMA hydrogel films, and grooved GelMA-MXene hydrogel films, determined by RT-qPCR assay. ∗p *<*0.05, ∗∗p *<*0.01, ∗∗∗p *<*0.001 and ns means no significant difference.

**
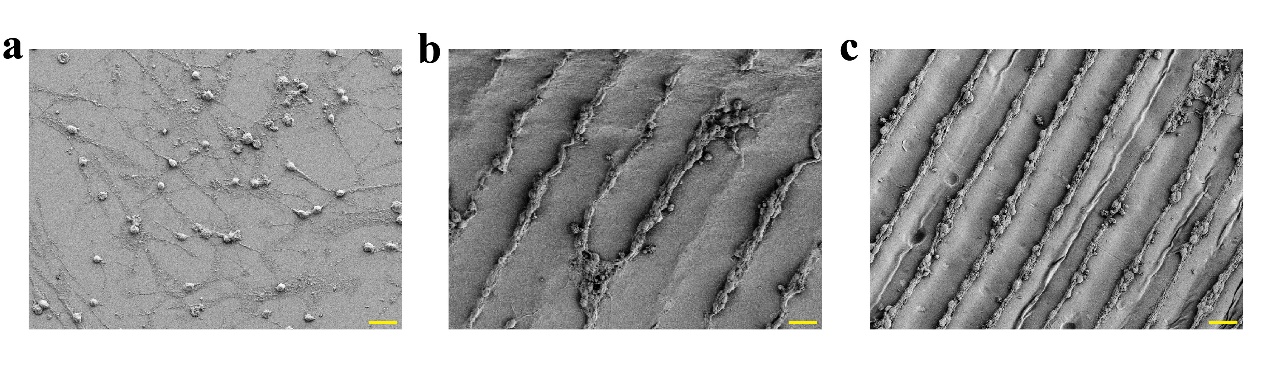
**

**Figure S5.** Apparent morphology NSCs differentiation on the three substrates. (a-c) SEM images of NSCs cultured on TCPs (a), grooved GelMA hydrogel films (b), and grooved GelMA-MXene hydrogel films (c) under differentiation medium for 3 d, respectively.


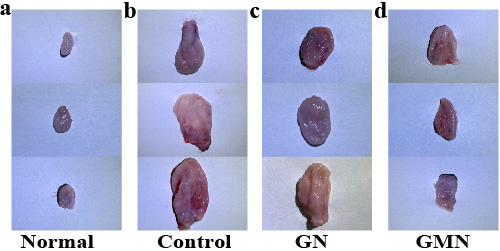


**Figure S6.** The bladders of different groups on week 8.

**
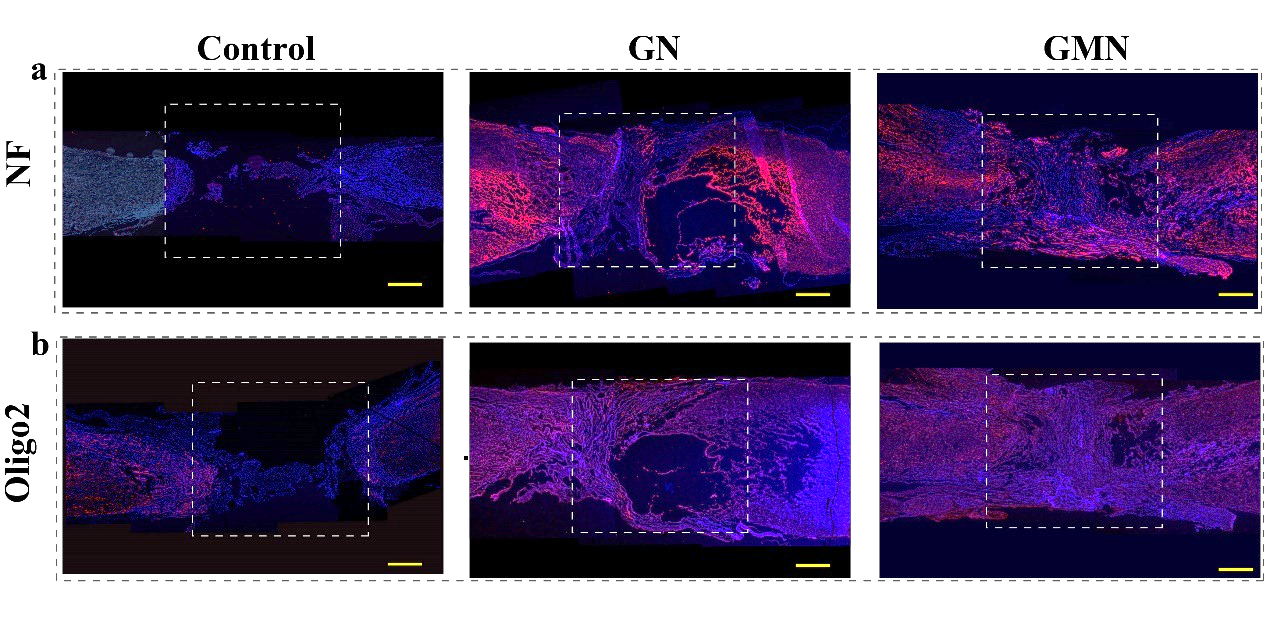
**

**Figure S7.** Representative images of spinal cord at the lesion rate. (a)Representative images showing NF (NF, red) in control groups, GN groups, GMN groups. (b) Representative images showing Oligo2 (Oligo2, red) in control groups, GN groups, GMN groups.


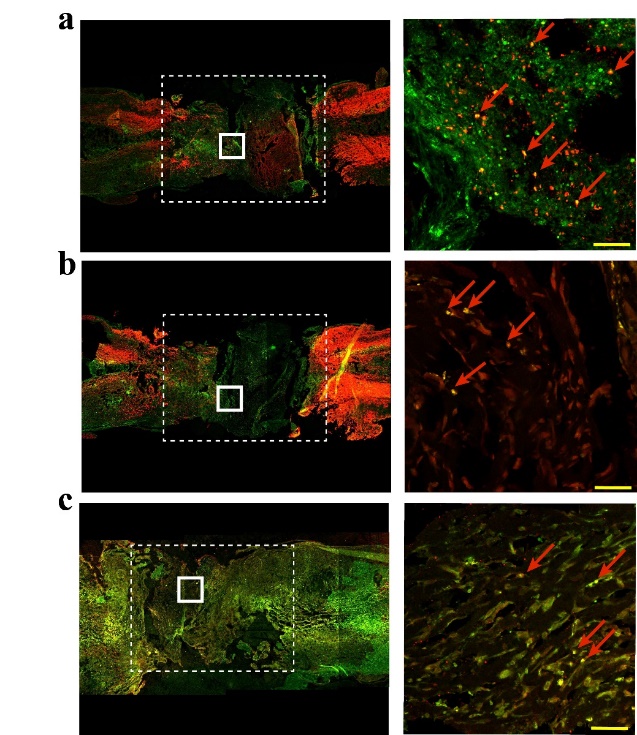


**Figure S8.** Survival and differentiation of the grafted NSCs in the lesion site four weeks post-implantation. (a) Representative images showing Tuj-1 (red) and GFP (green); (b) Representative images showing NF (red) and GFP (green). (c) Representative images showing Oligo2 (red) and GFP (green). The white dotted box shows the lesion site, and the red arrow illustrates the colocalization.
